# Adaptive Neural Control for Constrained Biomimetic Rehabilitation Robots Using a Novel High-Order Integral Barrier Function

**DOI:** 10.3390/biomimetics11080536

**Published:** 2026-08-02

**Authors:** Tan Zhang, Jinzhong Zhang, Pianpian Yan

**Affiliations:** 1 School of Electrical and Photoelectronic Engineering, West Anhui University, Lu’an 237012, China; 42000028@wxc.edu.cn (T.Z.); zhangjinzhongz@126.com (J.Z.); 2 Anhui Undergrowth Crop Intelligent Equipment Engineering Research Center, Lu’an 237012, China; 3 School of Electronics and Information Engineering, West Anhui University, Lu’an 237012, China

**Keywords:** biomimetic rehabilitation robot, tracking control, neural networks (NNs), high-order integral barrier function, joint error constraints, model uncertainty

## Abstract

To address the challenges of lumped model uncertainties and tracking error constraints in biomimetic rehabilitation robot control, this paper proposes a novel high-order integral barrier function to construct an adaptive neural tracking control scheme. Radial basis function neural networks (NNs), inspired by the receptive field mechanism of motor neurons, feature local activation and can accurately approximate the nonlinear dynamics of such bionic rehabilitation devices. Distinct from traditional integral barrier Lyapunov functions, the presented high-order integral barrier function can accommodate both time-varying and time-invariant error constraints, while simplifying the controller derivation and ensuring full differentiability of virtual control laws throughout the backstepping framework. Supported by the derived barrier function theorems, the tracking error of the robot is theoretically proven to stay within predefined safe boundaries and converge exponentially to a compact neighborhood of the origin. Finally, comparative numerical simulations on a biomimetic rehabilitation robot validate the effectiveness of the proposed theorem and constrained adaptive neural control strategy.

## 1. Introduction

Tracking control constitutes the core of functional realization for biomimetic rehabilitation robots, and its tracking performance directly determines the rehabilitation effect and practical application scope of such robotic devices. In rehabilitation therapy scenarios, smooth joint trajectory tracking is essential to guarantee safe human–robot interaction and improve the recovery efficiency of patients with motor dysfunction. It can be concluded that biomimetic rehabilitation robots are incapable of executing complex rehabilitation training tasks in practical environments without stable and high-precision tracking control. Over the past two decades, the control of robotic manipulators has attracted extensive research attention, and a variety of advanced motion control approaches have been proposed by numerous scholars [1,2,3,4,5].

Sliding mode control, as a representative nonlinear control strategy in the field of robust control, has undergone decades of theoretical iterations, evolving continuously from classical linear sliding mode to terminal sliding mode, high-order sliding mode, etc. Sliding mode control has become the mainstream technology choice for dealing with complex and uncertain system control problems, such as robotic manipulators [6,7,8], and also provides a practical approach for high-precision stable control in various strongly nonlinear scenarios. The backstepping control method provides a nonlinear design approach to the tracking control of manipulators [9,10,11]. It decomposes complex high-order dynamic systems into multiple low-order submodules, constructs virtual control variables step by step, and derives stability conditions recursively based on Lyapunov theory, thereby ensuring the global stability of the closed-loop system. Radial basis function neural networks (NNs) originate from the local receptive response mechanism of biological motor neurons. As such, they function as bio-inspired universal approximators, with the capability to fit arbitrary nonlinear functions and conduct online self-learning. This characteristic supports real-time approximation and compensation of hard-to-model lumped uncertain dynamics without pre-acquiring a complete and accurate dynamic model of robotic manipulators [12,13,14]. NNs provide a distinctive robust adaptive strategy to overcome the limitation of model dependence in the high-precision control of robotic manipulators. Fuzzy logic control constructs a rule-based mapping framework based on fuzzy set theory. It eliminates the requirement for prior acquisition of the manipulator’s precise dynamic model [15,16]. Relying solely on fuzzy rules extracted from accumulated practical experience in joint motion control, the control scheme realizes high-precision online approximation and compensation for lumped uncertain dynamics in manipulator systems. The aforementioned sliding mode control, backstepping strategy, neural network approximation and fuzzy adaptive control mainly focus on suppressing system uncertainties and improving tracking accuracy, while they seldom take into account mandatory limits on joint tracking errors induced by the safety protection needs of rehabilitation robots.

For biomimetic rehabilitation robots in physical human–robot interaction tasks, fixed joint error thresholds cannot fully meet the practical safety requirements during actual rehabilitation training. The mechanical impedance of the human limb varies throughout the training process under the influence of muscle activation, passive stretch resistance and different rehabilitation training phases. Such time-varying interactive characteristics have been verified in practical control studies, and ref. [17] developed an impedance learning hybrid adaptive controller for upper limb rehabilitation robots, which realized online learning of time-varying human–machine interactive impedance and verified the obvious time-varying feature of limb impedance in repeated rehabilitation motions through simulation and experimental tests. Constant error limits will either degrade the motion smoothness of the robotic device or result in excessive joint tracking deviation, which may cause uncomfortable stretching sensation on the user’s impaired limb. To tackle the safety risk caused by excessive joint tracking deviations in the rehabilitation process, ref. [18] designed an adaptive neural control scheme with output constraints for upper limb rehabilitation robots, which strictly limits tracking outputs within preset safe ranges to prevent excessive deviations from harming the patient’s impaired limb. Accordingly, time-varying joint error constraints that adapt the allowable error range to interactive conditions are required in practical rehabilitation applications, which urgently demands the development of advanced constrained control strategies for rehabilitation robots.

To satisfy the constraint requirements raised by human–robot interactive rehabilitation training, we first analyze the two fundamental constraint categories: state constraints and error constraints. In the motion control of biomimetic rehabilitation robots, state and error constraints serve as the core prerequisites for the safe and stable operation of the system. State constraints strictly limit parameters such as joint positions within a certain interval to avoid self-collision. Error constraints, on the other hand, confine end-effector tracking deviations within task-permitted thresholds, satisfying control accuracy specifications. The constrained control of these robots can significantly enhance system robustness, enabling these robots to simultaneously meet safety and precision demands under complex working conditions. The proposal of barrier Lyapunov functions provides a fruitful solution to constraint problems. Among them, the logarithmic barrier Lyapunov function can generate corresponding constraint repulsive forces when the constraint error approaches the boundary condition and achieve stable control under symmetric constant constraints [19], symmetric time-varying constraints [20], or asymmetric time-varying constraints [21]. The introduction of the integral barrier Lyapunov function delivers a targeted solution to the inherent limitations of logarithmic barrier methods [22,23,24]. Different from the conventional schemes that can only achieve state constraints by constraining tracking errors, this type of function directly embeds the system state boundaries into its own integral terms, which broadens the initial feasible region of the closed-loop system [25]. A representative alternative to the aforementioned barrier Lyapunov functions is the tangent-type one, which eliminates the requirement for extra inequality scaling preconditions during the stability proof of closed-loop control systems [26]. This feature not only reduces the theoretical derivation complexity of the constrained controller but also avoids the intrinsic conservativeness introduced by inequality scaling operations [27].

After decades of research efforts, various improved barrier Lyapunov functions have been developed. A bounded barrier Lyapunov function is proposed to address output tracking error constraints of space robots, which is integrated with radial basis function neural networks to form an adaptive control scheme [28]. An auxiliary system is introduced to cope with input saturation. The radial basis function neural network is utilized to approximate inertial parameter uncertainties and compensate joint input dead-zone nonlinearities simultaneously. Barrier Lyapunov functions with an exponentially decreasing barrier are used to obtain adaptive learning laws for differential neural network-based robotic identifiers [29]. This barrier function yields bounded identification errors with predefined exponential decay, and guarantees the convergence of neural network weights to the best fit values. Validation is carried out on the virtual Cartesian robot model and the actual Kinova Gen3 Lite manipulator working under haptic mode. Apart from barrier Lyapunov functions, another mainstream constraint guarantee tool named control barrier functions has also been widely studied for system safety limitation. To guarantee state safety constraints and avoid constraint violation during system operation, researchers have developed control barrier functions. Quadratic-program-based control Lyapunov-barrier function (QP-CLBF) is unified with filtering-based concurrent learning adaptive laws to cope with parametric uncertainties and unknown control coefficients of nonlinear systems [30]. The unified adaptive QP-CLBF achieves exponential convergence of unknown parameters and secures the maximal safe region without numerical state derivative estimation. Lyapunov theoretical proof and numerical simulations are conducted to verify the derived results. Control barrier functions are adopted to guarantee forward set invariance so that robots can complete long-term persistent tasks without battery depletion [31]. The battery non-depletion constraint is formulated as forward invariance conditions, and the effectiveness is verified by simulations and physical experiments of multi-robot area coverage missions. For manipulator-specific joint motion safety constraints, scholars further optimized control barrier function frameworks against model inaccuracies. Existing control barrier function schemes for manipulators rely on accurate system models or conservative worst-case uncertainty assumptions [32]. Zeroing control barrier functions are constructed to enforce joint position and velocity constraints, and online historical data are adopted to narrow the bounds of unknown parameters and decrease the conservatism of robust adaptive control barrier functions. The feasibility of this data-driven composite learning strategy is verified on manipulator platforms. In addition to safety constraint control, predefined-time fault-tolerant control for actuator faults has been studied for robotic systems. A zero-sum differential game based predefined-time optimal fault-tolerant control scheme is developed for space robots with actuator faults using adaptive dynamic programming [33]. Actuator faults are treated as one player in the zero-sum differential game, and the Hamilton–Jacobi–Isaacs equation is approximated by a single critic neural network. Predefined-time stability is proven via Lyapunov theory, and simulations verify the fault-tolerant tracking performance.

However, the three typical barrier functions mentioned above all have clear application limitations. The logarithmic barrier function can adapt to both symmetric and asymmetric constraint cases, but it cannot be used to design a stable controller for unconstrained systems. Although the typical integral barrier function can stabilize unconstrained systems, it cannot handle asymmetric constraint problems. It is restricted by the target value in the integral term and thus cannot be applied to the prescribed performance control of tracking error, and this target value additionally increases the design complexity of the controller. In the process of designing control systems based on the classical tangent-type barrier Lyapunov function, although this method does not require additional inequality conditions to prove the stability of the closed-loop systems, it does not have the capability to handle general asymmetric constraints, and the tangent barrier Lyapunov function cannot directly adapt to the constraint processing requirements of high-order systems. It should be emphasized that the above asymmetric constraint processing capacity only belongs to the logarithmic barrier function, which is not taken as an optimization target of our improved method. Based on the above, a universal high-order integral barrier function is coined in this paper to eliminate the drawbacks that logarithmic barrier functions cannot be implemented in controller design for unconstrained systems, existing integral barrier functions are not suitable for prescribed performance control, and tangent barrier functions cannot be further extended to high-order forms. The distinct novelties of this study are elaborated in the following.

- A novel universal high-order integral barrier function is coined in this work to reduce the complexity when existing integral barrier functions are utilized in controller design [22,23,24], and remedy the issues that tangent barrier functions cannot be extended to high-order forms [26,27], while logarithmic barrier functions are inapplicable to controller design for unconstrained systems [34,35].
- The coined universal high-order integral barrier function has adjustable parameters. With the help of these parameters, the constrained force intensity of the barrier function can be flexibly adjusted to adapt to the safety boundary requirements of biomimetic rehabilitation robots in different scenarios, prevent the system state from crossing the boundary, and balance control accuracy and convergence speed simultaneously.
- Corresponding theorems for the coined novel universal high-order integral barrier function are presented to verify the stability of the biomimetic rehabilitation robot control system. In addition, neural networks are incorporated in this study to handle the system’s unmodeled dynamics.

## 2. Preliminaries

### 2.1. Mathematical Model of Biomimetic Rehabilitation Robotic Manipulators

The mathematical description of the biomimetic rehabilitation robot employed in [12,35] is given as follows:

(1) $$M_{0}\left(q\right)\ddot{q}+C\left(q,\dot{q}\right)\dot{q}+G\left(q\right)=\tau \left(t\right)-J^{T}\left(q\right)f\left(t\right)$$

where $q\in R^{n}$ stands for the biomimetic rehabilitation robot’s joint position, so the first derivative and second derivative of *q* mean velocity and acceleration, respectively. In order to facilitate the inference of the controller, the mathematical description (1) is rewritten as follows:

(2) $$\left\{\begin{array}{c} {\dot{x}}_{1}=x_{2}\\ {\dot{x}}_{2}=M_{0}^{-1}\left(x_{1}\right)\left(\tau \left(t\right)-M_{un}\right) \end{array}\right.$$

where $x_{1}=q$ and $x_{2}=\dot{q}$, $M_{un}=C\left(x_{1},x_{2}\right)x_{2}+G\left(x_{1}\right)+J^{T}\left(x_{1}\right)f\left(t\right)$ with $M_{0}\in R^{n\times n}$ meaning a known symmetric positive-definite inertia matrix and ${\dot{M}}_{0}\left(x_{1}\right)-2C\left(x_{1},x_{2}\right)$ being skew-symmetirc, $J\in R^{n\times n}$ being the Jacobian matrix, $f\left(t\right)\in R^{n}$ representing the uncertain external force, $C\in R^{n\times n}$ and $G\in R^{n}$ expressing the unknown centripetal and Coriolis matrix and gravitational vector, respectively.

### 2.2. Universal Integral Barrier Function

**Definition 1.**  *A novel universal integral barrier function* $V_{0}$ *is coined as shown below:*

(3) $$V_{0}=\int_{0}^{z}\frac{pk_{b}^{2q}\left(t\right)\delta^{2q-1}}{k_{b}^{2q}\left(t\right)-\delta^{2q}}d\delta ,\;\left|z\right|<k_{b}\left(t\right)$$

*where* q≥1 *is a positive integer,* p>0 *is an adjustable parameter, z means the constrained error,* $k_{b}\left(t\right)>0$ *expresses the constraint boundary, and δ stands for the integration variable.*

**Theorem 1.**  *In the set* $\Omega_{0}=\left\{z:\left|z\right|<k_{b}\left(t\right)\right\}$*, the function* $V_{0}$ *is continuously differentiable, positive definite, and* $V_{0}\to \infty$ *as* $\left|z\right|\to k_{b}\left(t\right)$*, that is,* $V_{0}$ *is an effective barrier function.*

**Proof of Theorem 1.**  In the set $\Omega_{0}=\left\{z:\left|z\right|<k_{b}\left(t\right)\right\}$, let $L_{1}\left(z\right)=\int_{0}^{z}\frac{pk_{b}^{2q}\left(t\right)\delta^{2q-1}}{k_{b}^{2q}\left(t\right)-\delta^{2q}}d\delta -\frac{pz^{2q}}{2q}$, then the partial derivative of $L_{1}\left(z\right)$ with respect to *z* can be derived as $\frac{\partial L_{1}\left(z\right)}{\partial z}=\frac{pk_{b}^{2q}\left(t\right)z^{2q-1}}{k_{b}^{2q}\left(t\right)-z^{2q}}-pz^{2q-1}=\frac{pz^{4q-1}}{k_{b}^{2q}\left(t\right)-z^{2q}}$. It is easily observed that $\partial L_{1}\left(z\right)/\partial z>0$ as z>0 and $\partial L_{1}\left(z\right)/\partial z<0$ as z<0 owing to q≥1 is a positive integer. Moreover, the equation $\lim_{z\to 0^{+}}\frac{\partial L_{1}\left(z\right)}{\partial z}=\lim_{z\to 0^{-}}\frac{\partial L_{1}\left(z\right)}{\partial z}=0$ and $L_{1}\left(0\right)=0$ can be obtained, thereby $V_{0}=\int_{0}^{z}\frac{pk_{b}^{2q}\left(t\right)\delta^{2q-1}}{k_{b}^{2q}\left(t\right)-\delta^{2q}}d\delta \geq \frac{pz^{2q}}{2q}>0,\;z\neq 0$ and $V_{0}=0,\;z=0$ are true; that is, the function $V_{0}$ is positive definite. According to $\lim_{z\to 0^{+}}\frac{\partial V_{0}}{\partial z}=\lim_{z\to 0^{-}}\frac{\partial V_{0}}{\partial z}=0$, the function $V_{0}$ is continuously differentiable. By observing the function $V_{0}$, it can be found that the integrand tends to infinity when *z* approaches $k_{b}\left(t\right)$; hence, $V_{0}$ tends to infinity. □

**Theorem 2.**  *In the set* $\Omega_{0}=\left\{z:\left|z\right|<k_{b}\left(t\right)\right\}$*, the function* $V_{0}$ *obeys the following inequality:*

(4) $$\frac{pz^{2q}}{2q}\leq V_{0}=\int_{0}^{z}\frac{pk_{b}^{2q}\left(t\right)\delta^{2q-1}}{k_{b}^{2q}\left(t\right)-\delta^{2q}}d\delta \leq \frac{p}{2q}\frac{k_{b}^{2q}\left(t\right)z^{2q}}{k_{b}^{2q}\left(t\right)-z^{2q}}$$

**Proof of Theorem 2.**  In Theorem 1, the inequality $V_{0}\geq \frac{pz^{2q}}{2q}$ has been proven. Now, the second equation is defined as $L_{2}\left(z\right)=\frac{p}{2q}\frac{k_{b}^{2q}\left(t\right)z^{2q}}{k_{b}^{2q}\left(t\right)-z^{2q}}-\int_{0}^{z}\frac{pk_{b}^{2q}\left(t\right)\delta^{2q-1}}{k_{b}^{2q}\left(t\right)-\delta^{2q}}d\delta$. Then, calculating the partial derivative of $L_{2}\left(z\right)$, yields $\frac{\partial L_{2}\left(z\right)}{\partial z}=\frac{pk_{b}^{2q}\left(t\right)z^{4q-1}}{{\left(k_{b}^{2q}\left(t\right)-z^{2q}\right)}^{2}}$. It can be concluded that $\partial L_{2}\left(z\right)/\partial z>0$, z>0 and $\partial L_{2}\left(z\right)/\partial z<0$, z<0 because of q≥1 is a positive integer. Furthermore, the equation $\lim_{z\to 0^{+}}\frac{\partial L_{2}\left(z\right)}{\partial z}=\lim_{z\to 0^{-}}\frac{\partial L_{2}\left(z\right)}{\partial z}=0$ and $L_{2}\left(0\right)=0$ can be achieved, thus $V_{0}=\int_{0}^{z}\frac{pk_{b}^{2q}\left(t\right)\delta^{2q-1}}{k_{b}^{2q}\left(t\right)-\delta^{2q}}d\delta \leq \frac{p}{2q}\frac{k_{b}^{2q}\left(t\right)z^{2q}}{k_{b}^{2q}\left(t\right)-z^{2q}}$ holds. □

**Remark 1.** 
*According to the inequality (4) in Theorem 2, we can get* $\lim_{k_{b}\to \infty }\frac{p}{2q}\frac{k_{b}^{2q}\left(t\right)z^{2q}}{k_{b}^{2q}\left(t\right)-z^{2q}}=\frac{pz^{2q}}{2q}$*. Utilizing the squeeze theorem, we can deduce that* $\lim_{k_{b}\to \infty }V_{0}=\frac{pz^{2q}}{2q}$*. Therefore, the coined barrier function can be turned into a general quadratic form when* q=1*. This means that the devised barrier function can be employed to stabilize unconstrained systems. However, the high-order integral barrier function* $V_{pre}=\int_{0}^{N_{P}}\frac{\varepsilon \vartheta^{2p-1}}{1-\vartheta^{2p-1}}d\vartheta$ *coined by the author in previous literature [36] can only handle systems with constrained requirements because the barrier function* $V_{pre}$ *tends to 0 when the boundary conditions tend to infinity.*

**Remark 2.** 
*Although the traditional integral barrier function [22,23,24] can be used to handle unconstrained systems, it cannot be utilized for achieving prescribed performance control problems owing to the presence of target values in the integrand. Furthermore, compared with the traditional integral barrier function, the coined high-order barrier function can effectively ensure the differentiability of the virtual control law in backstepping control [35]. It should be noted that the integral barrier function previously coined by the author must normalize the error when constraining it [36].*

The biomimetic rehabilitation robot’s tracking error is defined as follows to better elaborate on the control objective of this study:

(5) $$\left\{\begin{array}{c} z_{1}={\left[z_{11},z_{12},\ldots ,z_{1n}\right]}^{T}=x_{1}-x_{d}\\ z_{2}={\left[z_{21},z_{22},\ldots ,z_{2n}\right]}^{T}=x_{2}-\alpha \end{array}\right.$$

where $x_{d}$ stands for the set trajectory, which is bounded and continuously differentiable, and α is the expected speed to be determined, which is also a virtual control law.

The neural network and universal high-order barrier function are used to coin an adaptive constraint control scheme for a biomimetic rehabilitation robot’s in the presence of system uncertainties. This control methodology aims to achieve the following **control objectives**: (i) The biomimetic rehabilitation manipulator can successfully track the set trajectory with the constructed controller. (ii) The closed-loop system error, containing the constrained error, can converge to a small neighborhood near zero exponentially. (iii) The constraints $-k_{bi}\left(t\right)<z_{1i}<k_{bi}\left(t\right)$, i=1,2,…,n on the biomimetic rehabilitation manipulator’s joint position errors are never broken. (iv) System uncertainties can be effectively approximated by neural networks.

## 3. Adaptive Constraint Controller Design

Under the backstepping framework, an adaptive-constraint tracking controller for biomimetic rehabilitation manipulators is coined utilizing neural networks and a first-put-forward high-order integral barrier function. The specific design process is as follows.

For the biomimetic rehabilitation manipulator, consider the following integral barrier function:

(6) $$V_{1}=\sum_{i=1}^{n}\int_{0}^{z_{1i}}\frac{p_{i}k_{bi}^{2q}\left(t\right)\delta^{2q-1}}{k_{bi}^{2q}\left(t\right)-\delta^{2q}}d\delta$$

where q≥1 is a positive integer, $p_{i}>0$ means an adjustable parameter.

In the set $\left|z_{1i}\right|<k_{bi}\left(t\right)$, i=1,2,…,n, give the derivative of $V_{1}$ with respect to time:


(7) $$\begin{array}{cc} {\dot{V}}_{1}= & \sum_{i=1}^{n}\frac{\partial V_{1}}{\partial z_{1i}}\frac{dz_{1i}}{dt}+\sum_{i=1}^{n}\frac{\partial V_{1}}{\partial k_{bi}\left(t\right)}\frac{dk_{bi}\left(t\right)}{dt}\\ = & \sum_{i=1}^{n}\frac{p_{i}k_{bi}^{2q}\left(t\right)z_{1i}^{2q-1}}{k_{bi}^{2q}\left(t\right)-z_{1i}^{2q}}{\dot{z}}_{1i}-\frac{p_{i}k_{bi}^{2q-1}\left(t\right)z_{1i}^{2q}}{k_{bi}^{2q}\left(t\right)-z_{1i}^{2q}}{\dot{k}}_{bi}\left(t\right)\\ \; & +p_{i}k_{bi}^{2q-1}\left(t\right)\log\left(\frac{k_{bi}^{2q}\left(t\right)}{k_{bi}^{2q}\left(t\right)-z_{1i}^{2q}}\right){\dot{k}}_{bi}\left(t\right) \end{array}$$

Utilizing the derivative of ${\dot{z}}_{1}=z_{2}+\alpha -{\dot{x}}_{d}$, (${\dot{z}}_{1i}=z_{2i}+\alpha_{i}-{\dot{x}}_{di}$) and the backstepping method, α is designed as:

(8) $$\begin{array}{cc} \alpha = & {\dot{x}}_{d}-I_{1}-I_{2}\\ \alpha_{i}= & {\dot{x}}_{di}-I_{1i}-I_{2i} \end{array}$$

where

(9) $$\begin{array}{c} I_{1}=\left[\begin{array}{c} I_{11}\\ \begin{array}{c} I_{12}\\ \cdots \\ I_{1n} \end{array} \end{array}\right]=\left[\begin{array}{c} \beta_{11}z_{11}\\ \begin{array}{c} \beta_{12}z_{12}\\ \cdots \\ \beta_{1n}z_{1n} \end{array} \end{array}\right] \end{array}$$

(10) $$\begin{array}{c} I_{2}={\left[I_{21},I_{22},\cdots ,I_{2n}\right]}^{T}=I_{diag}I_{dot}I^{\prime } \end{array}$$

with $\beta_{1i}$ being a positive constant, and

(11) $$\begin{array}{c} I_{diag}=\left[\begin{array}{cccc} \frac{k_{b1}^{2q}\left(t\right)-z_{11}^{2q}}{p_{1}k_{b1}^{2q}\left(t\right)} & & & \\ & \frac{k_{b2}^{2q}\left(t\right)-z_{12}^{2q}}{p_{2}k_{b2}^{2q}\left(t\right)} & & \\ & & \cdots & \\ & & & \frac{k_{bn}^{2q}\left(t\right)-z_{1n}^{2q}}{p_{n}k_{bn}^{2q}\left(t\right)} \end{array}\right] \end{array}$$

(12) $$\begin{array}{c} I^{\prime }=\left[\begin{array}{c} \left(\frac{k_{b1}^{2q-1}\left(t\right)}{z_{11}^{2q-1}}\log\frac{k_{b1}^{2q}\left(t\right)}{k_{b1}^{2q}\left(t\right)-z_{11}^{2q}}-\frac{k_{b1}^{2q-1}\left(t\right)z_{11}}{k_{b1}^{2q}\left(t\right)-z_{11}^{2q}}\right)\\ \begin{array}{c} \left(\frac{k_{b2}^{2q-1}\left(t\right)}{z_{12}^{2q-1}}\log\frac{k_{b2}^{2q}\left(t\right)}{k_{b2}^{2q}\left(t\right)-z_{12}^{2q}}-\frac{k_{b2}^{2q-1}\left(t\right)z_{12}}{k_{b2}^{2q}\left(t\right)-z_{12}^{2q}}\right)\\ \;\;\;\;\;\;\;\;\;\cdots \\ \left(\frac{k_{bn}^{2q-1}\left(t\right)}{z_{1n}^{2q-1}}\log\frac{k_{bn}^{2q}\left(t\right)}{k_{bn}^{2q}\left(t\right)-z_{1n}^{2q}}-\frac{k_{bn}^{2q-1}\left(t\right)z_{1n}}{k_{bn}^{2q}\left(t\right)-z_{1n}^{2q}}\right) \end{array} \end{array}\right] \end{array}$$

(13) $$\begin{array}{c} I_{dot}=\left[\begin{array}{cccc} p_{1}{\dot{k}}_{b1}\left(t\right) & & & \\ & p_{2}{\dot{k}}_{b2}\left(t\right) & & \\ & & \cdots & \\ & & & p_{n}{\dot{k}}_{bn}\left(t\right) \end{array}\right] \end{array}$$

**Remark 3.** 
*A singularity determination is required because the denominator in* $I^{\prime }$ *contains the error term* $z_{1i}$*. Through simple calculations, we can obtain* $\lim_{z_{1i}\to 0}\frac{k_{bi}^{2q-1}\left(t\right)}{z_{1i}^{2q-1}}\log\frac{k_{bi}^{2q}\left(t\right)}{k_{bi}^{2q}\left(t\right)-z_{1i}^{2q}}=\frac{2q}{2q-1}\frac{k_{bi}^{2q-1}\left(t\right)z_{1i}}{k_{bi}^{2q}\left(t\right)-z_{1i}^{2q}}$*. Therefore, the coined virtual control law α will not encounter singularity issues.*

According to (8)–(10), we know that $I_{1i}=\beta_{1i}z_{1i}$ and $I_{2i}=\frac{\left(k_{bi}^{2q}\left(t\right)-z_{1i}^{2q}\right){\dot{k}}_{bi}\left(t\right)}{z_{1i}^{2q-1}k_{bi}\left(t\right)}\log\frac{k_{bi}^{2q}\left(t\right)}{k_{bi}^{2q}\left(t\right)-z_{1i}^{2q}}-\frac{{\dot{k}}_{bi}\left(t\right)z_{1i}}{k_{bi}\left(t\right)}$. Substituting ${\dot{z}}_{1i}$ and $\alpha_{i}$ into ${\dot{V}}_{1}$ in turn yields:

(14) $$\begin{array}{cc} {\dot{V}}_{1}= & \sum_{i=1}^{n}\frac{p_{i}k_{bi}^{2q}\left(t\right)z_{1i}^{2q-1}}{k_{bi}^{2q}\left(t\right)-z_{1i}^{2q}}\left(z_{2i}-I_{1i}-I_{2i}\right)\\ \; & -\frac{p_{i}k_{bi}^{2q-1}\left(t\right)z_{1i}^{2q}}{k_{bi}^{2q}\left(t\right)-z_{1i}^{2q}}{\dot{k}}_{bi}\left(t\right)\\ \; & +p_{i}k_{bi}^{2q-1}\left(t\right)\log\left(\frac{k_{bi}^{2q}\left(t\right)}{k_{bi}^{2q}\left(t\right)-z_{1i}^{2q}}\right){\dot{k}}_{bi}\left(t\right)\\ = & \sum_{i=1}^{n}\frac{p_{i}k_{bi}^{2q}\left(t\right)z_{1i}^{2q-1}}{k_{bi}^{2q}\left(t\right)-z_{1i}^{2q}}\left(z_{2i}-\beta_{1i}z_{1i}-I_{2i}\right)\\ \; & -\frac{p_{i}k_{bi}^{2q-1}\left(t\right)z_{1i}^{2q}}{k_{bi}^{2q}\left(t\right)-z_{1i}^{2q}}{\dot{k}}_{bi}\left(t\right)\\ \; & +p_{i}k_{bi}^{2q-1}\left(t\right)\log\left(\frac{k_{bi}^{2q}\left(t\right)}{k_{bi}^{2q}\left(t\right)-z_{1i}^{2q}}\right){\dot{k}}_{bi}\left(t\right)\\ = & -\sum_{i=1}^{n}\frac{\beta_{1i}p_{i}k_{bi}^{2q}\left(t\right)z_{1i}^{2q}}{k_{bi}^{2q}\left(t\right)-z_{1i}^{2q}}+\sum_{i=1}^{n}\frac{p_{i}k_{bi}^{2q}\left(t\right)z_{1i}^{2q-1}}{k_{bi}^{2q}\left(t\right)-z_{1i}^{2q}}z_{2i}\\ \; & -\sum_{i=1}^{n}\frac{p_{i}k_{bi}^{2q}\left(t\right)z_{1i}^{2q-1}}{k_{bi}^{2q}\left(t\right)-z_{1i}^{2q}}I_{2i}-\frac{p_{i}k_{bi}^{2q-1}\left(t\right)z_{1i}^{2q}}{k_{bi}^{2q}\left(t\right)-z_{1i}^{2q}}{\dot{k}}_{bi}\left(t\right)\\ \; & +p_{i}k_{bi}^{2q-1}\left(t\right)\log\left(\frac{k_{bi}^{2q}\left(t\right)}{k_{bi}^{2q}\left(t\right)-z_{1i}^{2q}}\right){\dot{k}}_{bi}\left(t\right)\\ = & -\sum_{i=1}^{n}\frac{\beta_{1i}p_{i}k_{bi}^{2q}\left(t\right)z_{1i}^{2q}}{k_{bi}^{2q}\left(t\right)-z_{1i}^{2q}}+\sum_{i=1}^{n}\frac{p_{i}k_{bi}^{2q}\left(t\right)z_{1i}^{2q-1}}{k_{bi}^{2q}\left(t\right)-z_{1i}^{2q}}z_{2i} \end{array}$$

Given the second Lyapunov function:


(15) $$\begin{array}{c} V_{2}=V_{1}+\frac{1}{2}z_{2}^{T}M_{0}z_{2} \end{array}$$

Take ${\dot{z}}_{2}=M_{0}^{-1}\left(x_{1}\right)\left(\tau \left(t\right)-M_{un}\right)-\dot{\alpha }$ into the derivative of function $V_{2}$:

(16) $$\begin{array}{cc} {\dot{V}}_{2}= & \sum_{i=1}^{n}\frac{p_{i}k_{bi}^{2q}\left(t\right)z_{1i}^{2q-1}}{k_{bi}^{2q}\left(t\right)-z_{1i}^{2q}}z_{2i}-\sum_{i=1}^{n}\frac{\beta_{1i}p_{i}k_{bi}^{2q}\left(t\right)z_{1i}^{2q}}{k_{bi}^{2q}\left(t\right)-z_{1i}^{2q}}\\ \; & +z_{2}^{T}\left(\tau \left(t\right)-{M^{\prime }}_{un}-M_{0}\dot{\alpha }\right) \end{array}$$

where $M_{un}^{\prime }=C\left(x_{1},x_{2}\right)\alpha +G\left(x_{1}\right)+J^{T}\left(x_{1}\right)f\left(t\right)$ is unknown.

The bio-inspired radial basis function NNs are adopted to approximate the lumped unknown term $M_{un}^{\prime }$, and the adaptive constrained controller is designed as

(17) $$\begin{array}{c} \tau \left(t\right)={\hat{W}}^{T}S\left(In\right)-\beta_{2}z_{2}-\tau_{barrier}+M_{0}\dot{\alpha } \end{array}$$

where $\hat{W}$ means the fitting value of the ideal weight $W^{*}$, S expresses the basis function, and weight deviation is expressed by $\tilde{W}=\hat{W}-W^{*}$. Then the unknown term $M_{un}^{\prime }$ can be described as

(18) $$\begin{array}{c} M_{un}^{\prime }=W^{*T}S\left(In\right)+\omega \left(In\right) \end{array}$$

with $In=\left[x_{1}^{T},x_{2}^{T},\alpha^{T}\right]$ representing the input of the neural network. The fitting residual $\omega \left(In\right)$ obeys the inequality $\left|\omega \left(In\right)\right|<\omega_{b}$ with $\omega_{b}$ meaning a positive constant, $\beta_{2}=diag\left(\beta_{21},\beta_{22},\ldots ,\beta_{2n}\right)>0$, the adaption-update law ${\dot{\hat{W}}}_{i}$ and $\tau_{barrier}$ are coined as

(19) $$\begin{array}{c} {\dot{\hat{W}}}_{i}=-\Gamma_{i}\left(S_{i}\left(In\right)z_{2i}+\Theta_{i}{\hat{W}}_{i}\right) \end{array}$$

Gaussian radial basis functions are adopted as the activation functions of the network. $\Gamma_{i}\in R^{64\times 64}$ is a positive definite diagonal learning gain matrix, which adjusts the convergence rate of weight online updating. The term $\Theta_{i}{\hat{W}}_{i}$ included in Equation (19) is the σ-modification term, which suppresses weight parameter drift caused by long-term external disturbances. The neural network estimates the overall unknown dynamics of the manipulator. The fitting residual ω(In) supports the Lyapunov stability analysis.


(20) $$\begin{array}{c} \tau_{barrier}={\left[\frac{p_{1}k_{b1}^{2q}\left(t\right)z_{11}^{2q-1}}{k_{b1}^{2q}\left(t\right)-z_{11}^{2q}},\frac{p_{2}k_{b2}^{2q}\left(t\right)z_{12}^{2q-1}}{k_{b2}^{2q}\left(t\right)-z_{12}^{2q}},\ldots ,\frac{p_{n}k_{bn}^{2q}\left(t\right)z_{1n}^{2q-1}}{k_{bn}^{2q}\left(t\right)-z_{1n}^{2q}}\right]}^{T} \end{array}$$

**Theorem 3.** 
*For the biomimetic rehabilitation manipulator described mathematically as (2), if the joint position obeys* $\left|z_{1i}\left(0\right)\right|<k_{bi}\left(0\right)$*, the expected velocity is coined as (8), and the adaptive-constraint controller is given as (17) with the adaption-update law being (19), then the following conclusions are valid: (i) The biomimetic rehabilitation manipulator can track the desired trajectory, and the errors within the closed-loop system, including the constrained position errors, can converge exponentially to a neighborhood near the equilibrium point. (ii) The constraint condition* $\left|z_{1i}\left(t\right)\right|<k_{bi}\left(t\right)$*,* $t\in \left[0,+\infty \right)$*,* i=1,2,…,n*, which the joint position error follows, will not be violated.*

**Proof of Theorem 3.**  The candidate Lyapunov function for the closed-loop system is constructed as follows:

(21) $$\begin{array}{cc} V_{3} & =V_{2}+\frac{1}{2}\sum_{i=1}^{n}{\tilde{W}}_{i}^{T}\Gamma_{i}^{-1}{\tilde{W}}_{i}\\ \; & =\sum_{i=1}^{n}\int_{0}^{z_{1i}}\frac{p_{i}k_{bi}^{2q}\left(t\right)\delta^{2q-1}}{k_{bi}^{2q}\left(t\right)-\delta^{2q}}d\delta +\frac{1}{2}z_{2}^{T}M_{0}z_{2}+\frac{1}{2}\sum {\tilde{W}}_{i}^{T}\Gamma_{i}^{-1}{\tilde{W}}_{i} \end{array}$$

The Lyapunov function (21) consists of three components with clear physical meanings. The first integral term is constructed based on the integral barrier function. It constrains the joint position error $z_{1i}$ and ensures the time-varying safety constraint $|z_{1i}\left(t\right)|<k_{bi}\left(t\right)$ holds for all time. The second quadratic term of $z_{2}$ is associated with velocity tracking error $z_{2i}$, which is used to realize the convergence of closed-loop tracking error. The third term corresponds to the weight estimation error of the radial basis function neural network. Bounded weight error guarantees the approximation performance of unknown robotic dynamics, so that online compensation for system uncertainties can be realized.

After embedding the control law (17) into ${\dot{V}}_{3}$, the resulting expression is

(22) $$\begin{array}{cc} {\dot{V}}_{3}= & {\dot{V}}_{2}+\sum_{i=1}^{n}{\tilde{W}}_{i}^{T}\Gamma_{i}^{-1}{\dot{\hat{W}}}_{i}\\ = & \sum_{i=1}^{n}\frac{p_{i}k_{bi}^{2q}\left(t\right)z_{1i}^{2q-1}}{k_{bi}^{2q}\left(t\right)-z_{1i}^{2q}}z_{2i}-\sum_{i=1}^{n}\frac{\beta_{1i}p_{i}k_{bi}^{2q}\left(t\right)z_{1i}^{2q}}{k_{bi}^{2q}\left(t\right)-z_{1i}^{2q}}\\ \; & +z_{2}^{T}\left({\hat{W}}^{T}S\left(In\right)-\beta_{2}z_{2}-\tau_{barrier}-{M^{\prime }}_{un}\right)+\sum_{i=1}^{n}{\tilde{W}}_{i}^{T}\Gamma_{i}^{-1}{\dot{\hat{W}}}_{i}\\ = & -\sum_{i=1}^{n}\frac{\beta_{1i}p_{i}k_{bi}^{2q}\left(t\right)z_{1i}^{2q}}{k_{bi}^{2q}\left(t\right)-z_{1i}^{2q}}-\sum_{i=1}^{n}\beta_{2i}z_{2i}^{2}+\sum_{i=1}^{n}z_{2i}{\tilde{W}}_{i}^{T}S_{i}\left(In\right)\\ \; & -\sum_{i=1}^{n}z_{2i}\omega_{i}\left(In\right)+\sum_{i=1}^{n}{\tilde{W}}_{i}^{T}\Gamma_{i}^{-1}{\dot{\hat{W}}}_{i} \end{array}$$

Substitute ${\dot{\hat{W}}}_{i}$ into (22), and by virtue of Young’s inequality and Theorem 2, we arrive at:

(23) $$\begin{array}{cc} {\dot{V}}_{3}= & -\sum_{i=1}^{n}\frac{\beta_{1i}p_{i}k_{bi}^{2q}\left(t\right)z_{1i}^{2q}}{k_{bi}^{2q}\left(t\right)-z_{1i}^{2q}}-\sum_{i=1}^{n}\beta_{2i}z_{2i}^{2}\\ \; & -\sum_{i=1}^{n}z_{2i}\omega_{i}\left(In\right)-\sum_{i=1}^{n}\Theta_{i}{\tilde{W}}_{i}^{T}\left({\tilde{W}}_{i}+W_{i}^{*}\right)\\ \leq & -\sum_{i=1}^{n}\frac{\beta_{1i}p_{i}k_{bi}^{2q}\left(t\right)z_{1i}^{2q}}{k_{bi}^{2q}\left(t\right)-z_{1i}^{2q}}-z_{2}^{T}\left(\beta_{2}-\frac{1}{2}I\right)z_{2}\\ \; & -\frac{1}{2}\sum_{i=1}^{n}\Theta_{i}{\tilde{W}}_{i}^{T}{\tilde{W}}_{i}+\frac{1}{2}\sum_{i=1}^{n}\Theta_{i}{∥W_{i}^{*}∥}^{2}+\frac{1}{2}\omega_{b}^{2}\\ \leq & -\Lambda_{1}V_{3}+\Lambda_{2} \end{array}$$

where

(24) $$\begin{array}{cc} \Lambda_{1}= & \min_{i=1,2,\ldots ,n}\left(2q\beta_{1i},\frac{2\left(\beta_{2i}-\frac{1}{2}\right)}{\lambda_{\max}\left(M_{0}\right)},\frac{\Theta_{i}}{\lambda_{\max}\left(\Gamma_{i}^{-1}\right)}\right)\\ \Lambda_{2}= & \frac{1}{2}\sum_{i=1}^{n}\Theta_{i}{∥W_{i}^{*}∥}^{2}+\frac{1}{2}\omega_{b}^{2} \end{array}$$

with *I* meaning the identify matrix, $\lambda_{\max}\left(O\right)$ being the maximum eigenvalue of O, and $\beta_{1i}>0$, $\beta_{2i}>\frac{1}{2}$.

By resolving the inequality (23), the following result can be yielded:

(25) $$\begin{array}{c} 0\leq V_{3}\left(t\right)\leq V_{3}\left(0\right)e^{-\Lambda_{1}t}+\frac{\Lambda_{2}}{\Lambda_{1}}\overset{\Delta }{=}V_{\Lambda } \end{array}$$

Based on the coined Lyapunov function $V_{3}$, the subsequent inequalities hold naturally:

(26) $$\begin{array}{cc} \; & \int_{0}^{z_{1i}}\frac{p_{i}k_{bi}^{2q}\left(t\right)\delta^{2q-1}}{k_{bi}^{2q}\left(t\right)-\delta^{2q}}\leq V_{\Lambda }\\ \; & \frac{1}{2}z_{2}^{T}M_{0}z_{2}\leq V_{\Lambda }\\ \; & \frac{1}{2}\sum_{i=1}^{n}{\tilde{W}}_{i}^{T}\Gamma_{i}^{-1}{\tilde{W}}_{i}\leq V_{\Lambda } \end{array}$$

By resolving the pre-derived inequality (26), the following result is obtained:

(27) $$\begin{array}{c} z_{1i}^{2q}\leq k_{bi}^{2q}\left(1-e^{-\frac{2qV_{\Lambda }}{p_{i}k_{bi}^{2q}}}\right)\\ ∥z_{2}∥\leq \sqrt{2\left(V_{3}\left(t\right)e^{-\Lambda_{1}t}+\frac{\Lambda_{2}}{\Lambda_{1}}\right)/\lambda_{\min}(M_{0})}\\ ∥{\tilde{W}}_{i}∥\leq \sqrt{2\left(V_{3}\left(t\right)e^{-\Lambda_{1}t}+\frac{\Lambda_{2}}{\Lambda_{1}}\right)/\lambda_{\min}(\Gamma_{i}^{-1})} \end{array}$$

From the aforementioned derived inequality, $0<\left(1-e^{-\frac{2qV_{\Lambda }}{p_{i}k_{bi}^{2q}}}\right)<1$ can be directly validated to hold, which further indicates that $z_{1i}^{2q}<k_{bi}^{2q}$ and $\left|z_{1i}\right|<k_{bi}$. Subsequently, the inequality $\frac{p_{i}z_{1i}^{2q}}{2q}\leq V_{\Lambda }=V_{3}\left(0\right)e^{-\Lambda_{1}t}+\frac{\Lambda_{2}}{\Lambda_{1}}$ can be rigorously validated via the derived Theorem 2, which further deduces that $\left|z_{1i}\right|\leq {\left(\frac{2q}{p_{i}}\left(V_{3}\left(0\right)e^{-\Lambda_{1}t}+\frac{\Lambda_{2}}{\Lambda_{1}}\right)\right)}^{\frac{1}{2q}}$.

It can be summarized that the constrained position error stays strictly within the predefined constraint boundary for all time instants. Subsequently, by tuning proper parameters, all errors in the closed-loop system can converge exponentially to a small neighborhood around the equilibrium point. □

**Remark 4.** 
*From the existing constrained controllers for nonlinear systems coined with logarithmic and tangent barrier functions, it is observed that these controllers can only guarantee the boundedness of the constrained error [26,27,34,35]. By contrast, leveraging Theorem 2 derived in this work, the controller developed with the universal high-order barrier function can not only ensure the satisfaction of constraint conditions but also enable the constrained error to converge exponentially to a neighborhood around zero.*

**Remark 5.** 
*Drawing on the inequality* $\left|z_{1i}\right|\leq {\left(\frac{2q}{p_{i}}\left(V_{3}\left(0\right)e^{-\Lambda_{1}t}+\frac{\Lambda_{2}}{\Lambda_{1}}\right)\right)}^{\frac{1}{2q}}$ *derived in the proof of Theorem 3, it can be concluded that the adjustable parameters embedded in the coined universal high-order integral barrier function are capable of appropriately tuning the convergence precision of the constrained error, that is, a larger adjustable parameter* $p_{i}$ *will lead to a smaller tracking error* $z_{1i}$*.*

## 4. Comparative Simulations

**Figure 1:**
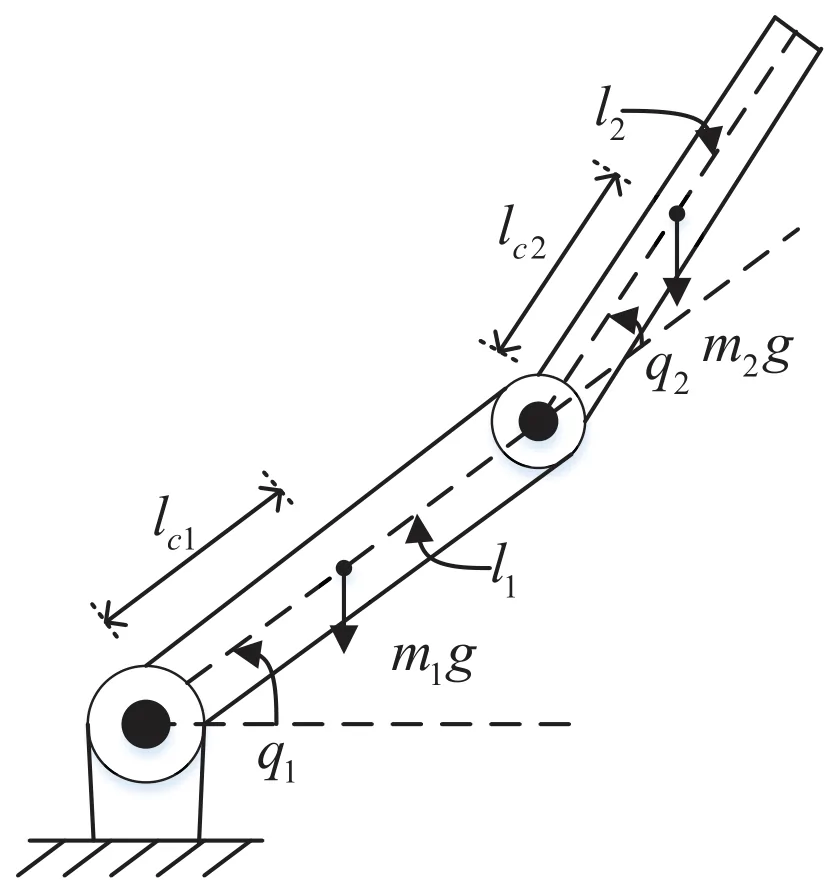
Kinematic schematic diagram of the dual-link manipulator.

We shall verify the control performance and practical operational effectiveness through comparative numerical simulations of a dual-link biomimetic rehabilitation manipulator. The developed controller integrates the universal high-order integral barrier function (UHIBF) and bio-inspired radial basis function NNs. The specific dynamic parameters of the dual-link manipulator model shown in Figure 1 used in the numerical simulation are presented below with reference values taken from [12,35].

(28) $$\begin{array}{c} M_{0}\left(q\right)=\left[\begin{array}{cc} M_{11} & M_{12}\\ M_{21} & M_{22} \end{array}\right] \end{array}$$

(29) $$\begin{array}{c} C\left(q,\dot{q}\right)=\left[\begin{array}{cc} C_{11} & C_{12}\\ C_{21} & C_{22} \end{array}\right] \end{array}$$

(30) $$\begin{array}{c} G\left(q\right)={\left[\begin{array}{cc} G_{11} & G_{12} \end{array}\right]}^{T} \end{array}$$

(31) $$\begin{array}{c} J\left(q\right)=\left[\begin{array}{cc} -\left(l_{1}\sin q_{1}+l_{2}\sin\left(q_{1}+q_{2}\right)\right) & -l_{2}\sin\left(q_{1}+q_{2}\right)\\ l_{1}\cos q_{1}+l_{2}\cos\left(q_{1}+q_{2}\right) & l_{2}\cos\left(q_{1}+q_{2}\right) \end{array}\right] \end{array}$$

with

(32) $$\begin{array}{cc} \; & M_{11}=m_{1}r_{1}^{2}+m_{2}\left(l_{1}^{2}+r_{2}^{2}+2l_{1}r_{2}\cos q_{2}\right)+I_{1}+I_{2}\\ \; & M_{12}=m_{2}\left(r_{2}^{2}+l_{1}r_{2}\cos q_{2}\right)+I_{2}\\ \; & M_{21}=m_{2}\left(r_{2}^{2}+l_{1}r_{2}\cos q_{2}\right)+I_{2}\\ \; & M_{22}=m_{2}r_{2}^{2}+I_{2}\\ \; & C_{11}=-m_{2}l_{1}r_{2}{\dot{q}}_{2}\sin q_{2}\\ \; & C_{12}=-m_{2}l_{1}r_{2}\left({\dot{q}}_{2}+{\dot{q}}_{1}\right)\sin q_{2}\\ \; & C_{21}=m_{2}l_{1}r_{2}{\dot{q}}_{1}\sin q_{2}\\ \; & C_{22}=0\\ \; & G_{11}=\left(m_{1}r_{2}+m_{2}l_{1}\right)g\cos q_{1}+m_{2}r_{2}g\cos\left(q_{1}+q_{2}\right)\\ \; & G_{12}=m_{2}r_{2}g\cos\left(q_{1}+q_{2}\right). \end{array}$$

where $l_{i}\left(i=1,2\right)$ and $m_{i}\left(i=1,2\right)$ denote the length and mass of the *i*-th link. It is assumed that equivalent masses are concentrated at the terminal end of every single link separately. $r_{i}\left(i=1,2\right)$ express the distance from joint i−1 to mass center of link *i*. The specific values of relevant parameters are given as: $m_{1}=2$ kg, $m_{2}=0.85$ kg, $l_{1}=0.35$ m, $l_{2}=0.31$ m, $I_{1}=61.25\times 10^{-3}$ kg m^2^ and $I_{2}=20.42\times 10^{-3}$ kg m^2^.

The reference trajectory used for numerical verification is set as follows:

(33) $$\begin{array}{c} x_{d}=\left[\begin{array}{c} 0.15sin\;(2t)\\ 0.15cos\;(2t) \end{array}\right] \end{array}$$

The constraint boundaries are set as:

(34) $$\begin{array}{c} k_{b1}=0.09+0.05cos\;\left(3t\right)\\ k_{b2}=0.09+0.05sin\;\left(3t\right) \end{array}$$

We assign $x_{1}={\left[0.1,0.1\right]}^{T}$ and $x_{2}={\left[0,0\right]}^{T}$ as the initial conditions of the biomimetic rehabilitation manipulator throughout the simulation, while the controller parameters are selected as $\beta_{11}=\beta_{12}=50$, $\beta_{21}=\beta_{22}=30$, q=2, $p_{1}=p_{2}=120$. We specify the unknown external disturbance acting on the biomimetic rehabilitation manipulator system as $f\left(t\right)={\left[6sin\;\left(6t\right)+20,4cos\;\left(0.5t\right)+30\right]}^{T}$. The following lists all configuration parameters of the bio-inspired radial basis function neural network adopted in simulation. The initial weight values are set to ${\hat{W}}_{1j}={\hat{W}}_{2j}=0,\;\left(j=1,2,\ldots ,64\right)$. The relevant network parameters are configured as $\Theta_{1}=0.0005$, $\Theta_{2}=0.000001$, and $\Gamma_{1}=\Gamma_{2}=15I_{64\times 64}$. $\Theta_{1}$ and $\Theta_{2}$ act as the coefficients of the σ-modification term embedded in the weight adaptive law, and $\Gamma_{1}$, $\Gamma_{2}$ represent the learning gain matrices for weight updating. The bio-inspired radial basis functions of NNs are selected as $S_{ij}\left(In\right)=exp\left(-{\left|In-\Phi_{j}\right|}^{2}/\varepsilon_{j}^{2}\right)$, where $\Phi_{j}$ are evenly spaced within the intervals [−0.15,0.15]×[−0.15,0.15]×[−0.15,0.15]×[−0.15,0.15]×[−0.15,0.15]×[−0.15,0.15] and $\varepsilon_{j}^{2}=25$, (i=1,2;j=1,2,…,64). Herein, $\Phi_{j}$ denotes the central points of Gaussian kernel functions, and $\varepsilon_{j}^{2}$ is the kernel width parameter.

For first comparison method, we employ the non-universal integral barrier function (NUIBF) developed in [37]. The observer utilized in this method cannot effectively estimate the unmodeled dynamics of the system. Accordingly, the biomimetic rehabilitation manipulator model is assumed to be fully known when implementing the control scheme mentioned above. This comparison condition helps highlight the superiority of our NNs in approximating model uncertainties. The tracking control strategy as well as the setting of the parameter values remains consistent with those in [37], as given below.

(35) $$\begin{array}{cc} \alpha = & {\dot{x}}_{d}-\left(K+\bar{K}\left(t\right)\right)z_{1}\\ \alpha_{i}= & {\dot{x}}_{di}-\left(k_{1i}+{\bar{k}}_{1i}\left(t\right)\right)z_{1i} \end{array}$$

where $K=diag(k_{11},k_{12})$, $\bar{K}\left(t\right)=diag\left({\bar{k}}_{11}\left(t\right),{\bar{k}}_{12}\left(t\right)\right)$ and ${\bar{k}}_{1i}\left(t\right)=\sqrt{{\left(\frac{{\dot{k}}_{bi}}{k_{bi}}\right)}^{2}+\epsilon_{i}}$ with $\epsilon_{i}>0$ and $k_{1i}>0$. The NUIBF-based controller takes the form:

(36) $$\begin{array}{c} \tau \left(t\right)=C\left(x_{1},x_{2}\right)x_{2}+G\left(x_{1}\right)+J^{T}\left(x_{1}\right)f\left(t\right)+M\alpha -K_{2}z_{2}-P_{\rho } \end{array}$$

where $P_{\rho }={\left[\frac{2Tr_{1}}{k_{b1}\left(1-Tr_{1}^{2}\right)},\frac{2Tr_{2}}{k_{b2}\left(1-Tr_{2}^{2}\right)}\right]}^{T}$ with $K_{2}=diag\left(k_{21},k_{22}\right)>0$ and $Tr_{1}=\frac{z_{1i}}{k_{bi}},\left(i=1,2\right)$.

For the second comparative experiment, we adopt the traditional logarithmic barrier function (LOGBF) utilized in [35]. Similar to the method proposed in this paper, neural networks are also employed to approximate and compensate for model uncertainties in the LOGBF-based control strategy. Furthermore, variables marked with the same symbols are assigned identical values across all three comparative methods in the simulation, so as to guarantee fair comparative simulations. The design of the virtual control law and controller τ(t) is given as follows.

(37) $$\begin{array}{cc} \; & \alpha ={\dot{x}}_{d}-\left(K+\bar{K}\left(t\right)\right)z_{1}\\ \; & \alpha_{i}={\dot{x}}_{di}-\left(K_{1i}+{\bar{K}}_{1i}\left(t\right)\right)z_{1i} \end{array}$$

(38) $$\begin{array}{c} \tau \left(t\right)={\hat{W}}^{T}S\left(In\right)-\beta_{2}z_{2}-\tau_{\log}+M_{0}\dot{\alpha } \end{array}$$

where the values of *K* and $\bar{K}\left(t\right)$ are consistent with those adopted in the NUIBF-based method. The value of $\beta_{2}$ remains the same as that used in the proposed method. The corresponding barrier term $\tau_{\log}$ is defined as $\tau_{\log}={\left[\frac{z_{11}}{k_{b1}^{2}-z_{11}^{2}},\frac{z_{12}}{k_{b2}^{2}-z_{12}^{2}}\right]}^{T}$.

**Figure 2:**
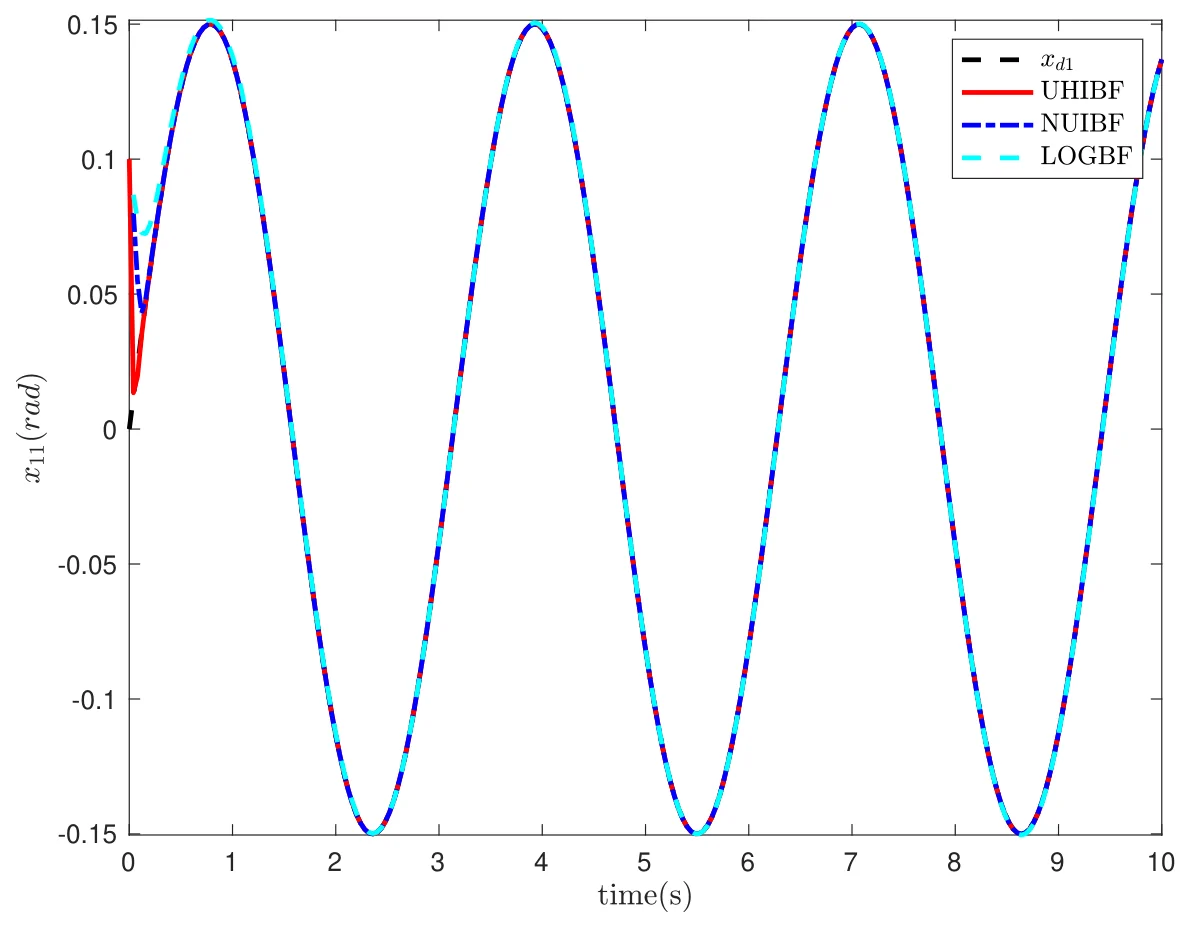
The curves of $x_{11}$ and $x_{d1}$.

**Figure 3:**
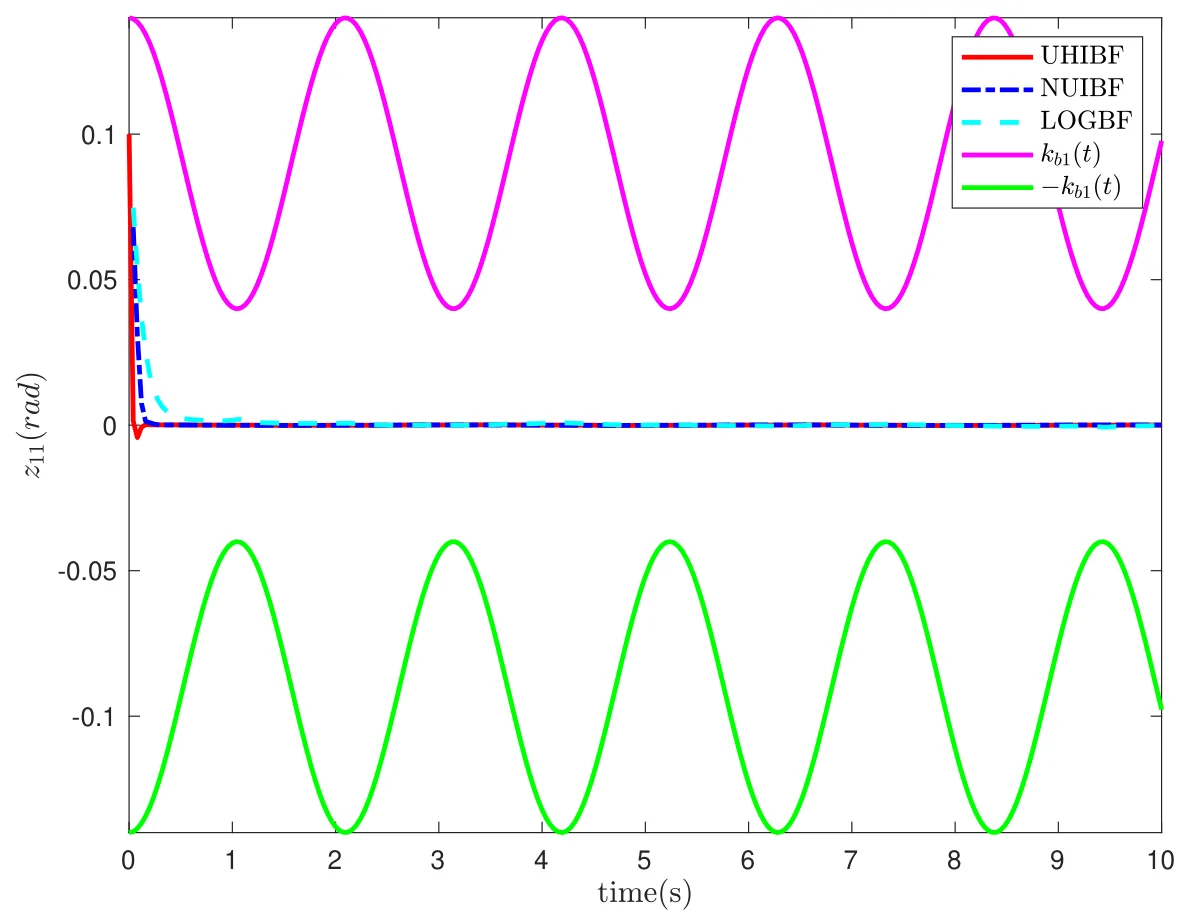
The curves of $z_{11}$.

**Figure 4:**
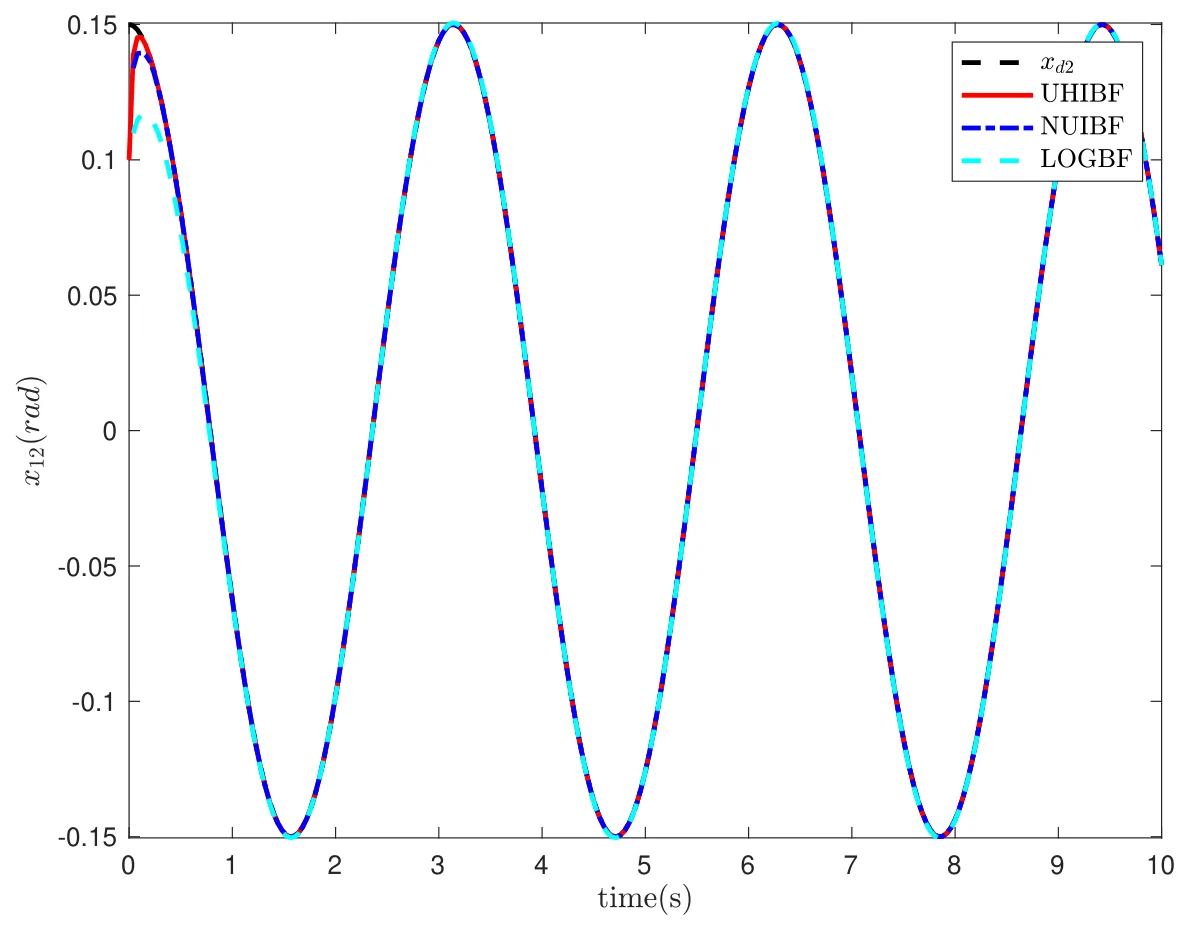
The curves of $x_{12}$ and $x_{d2}$.

**Figure 5:**
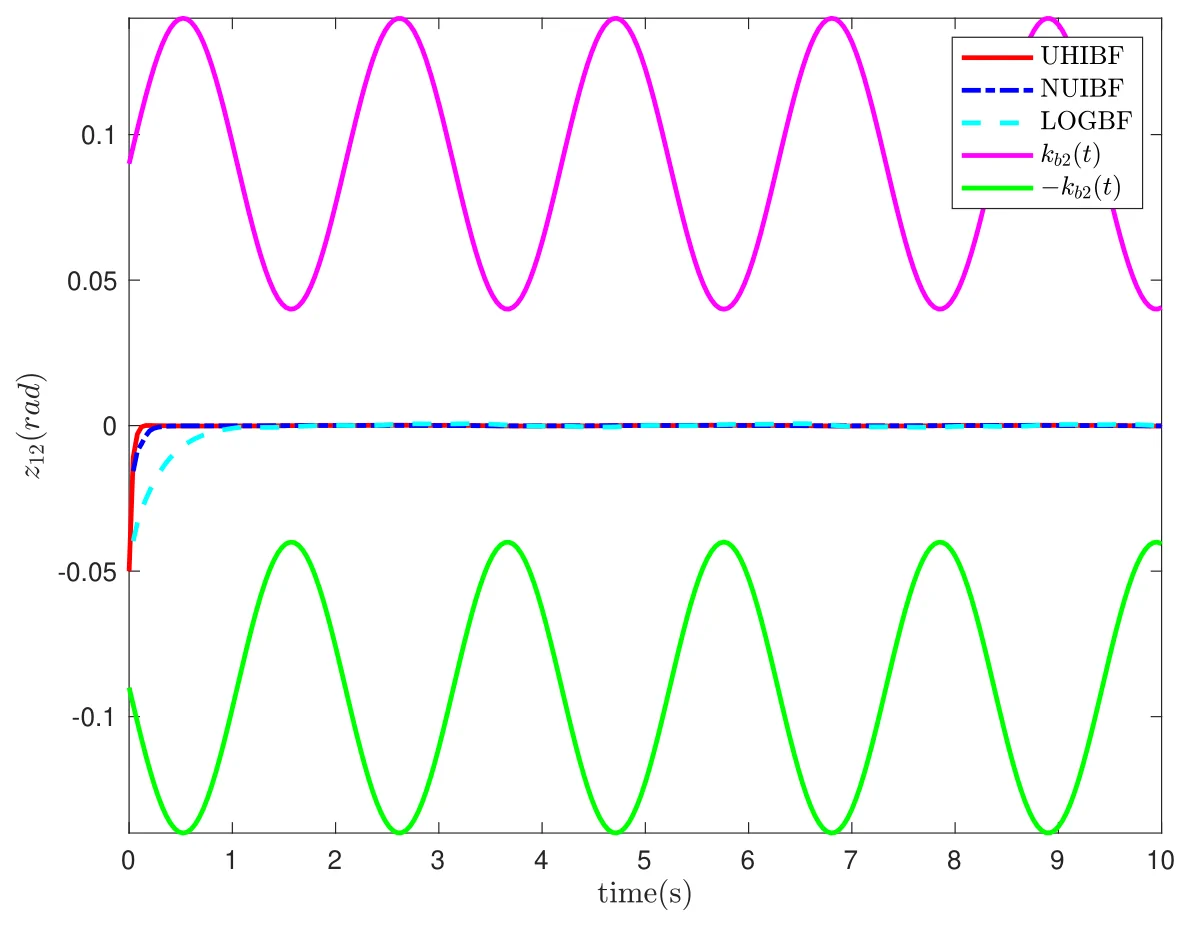
The curves of $z_{12}$.

**Figure 6:**
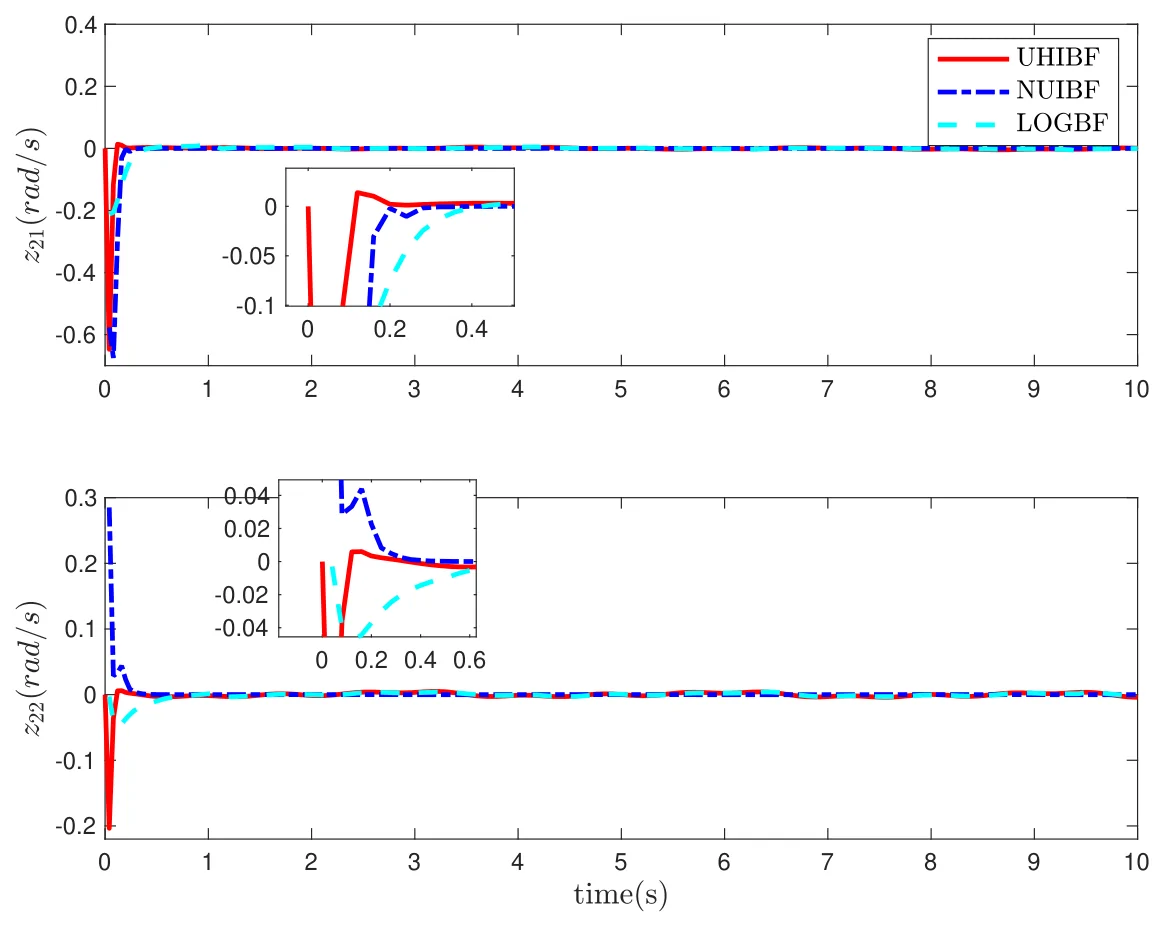
The curves of $z_{21}$ and $z_{22}$.

**Figure 7:**
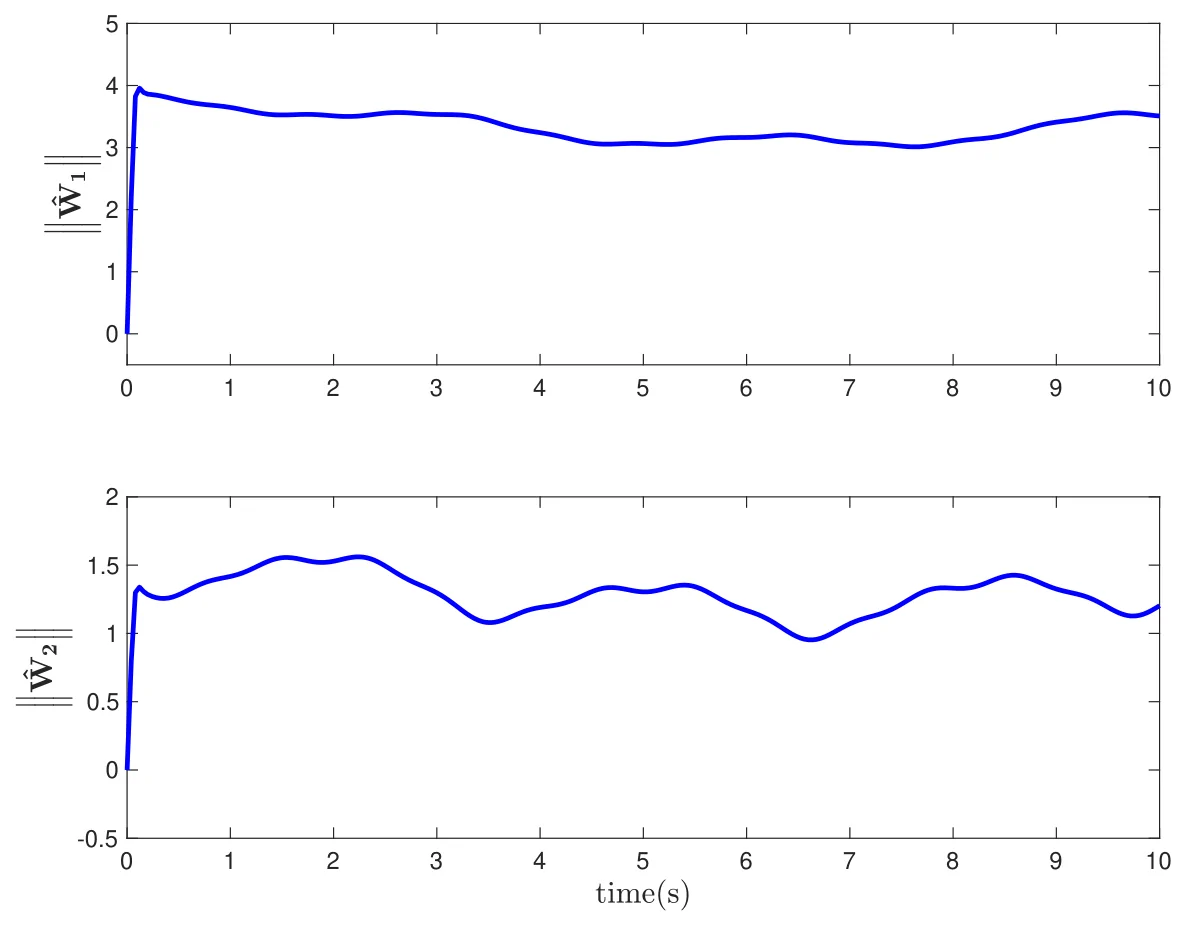
The curves of $∥{\hat{W}}_{1}∥$ and $∥{\hat{W}}_{2}∥$.

**Figure 8:**
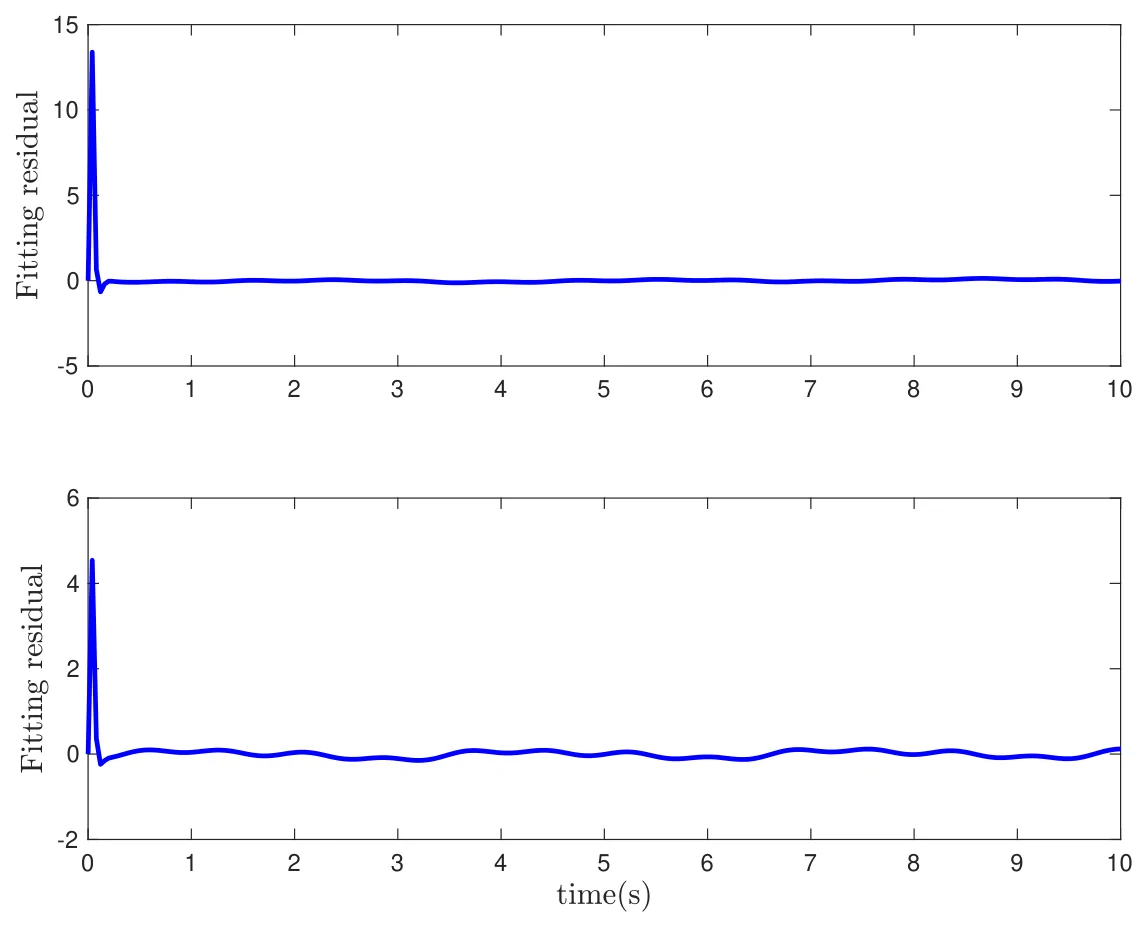
The fitting residual $M_{un}^{\prime }-{\hat{W}}^{T}S\left(In\right)$.

**Figure 9:**
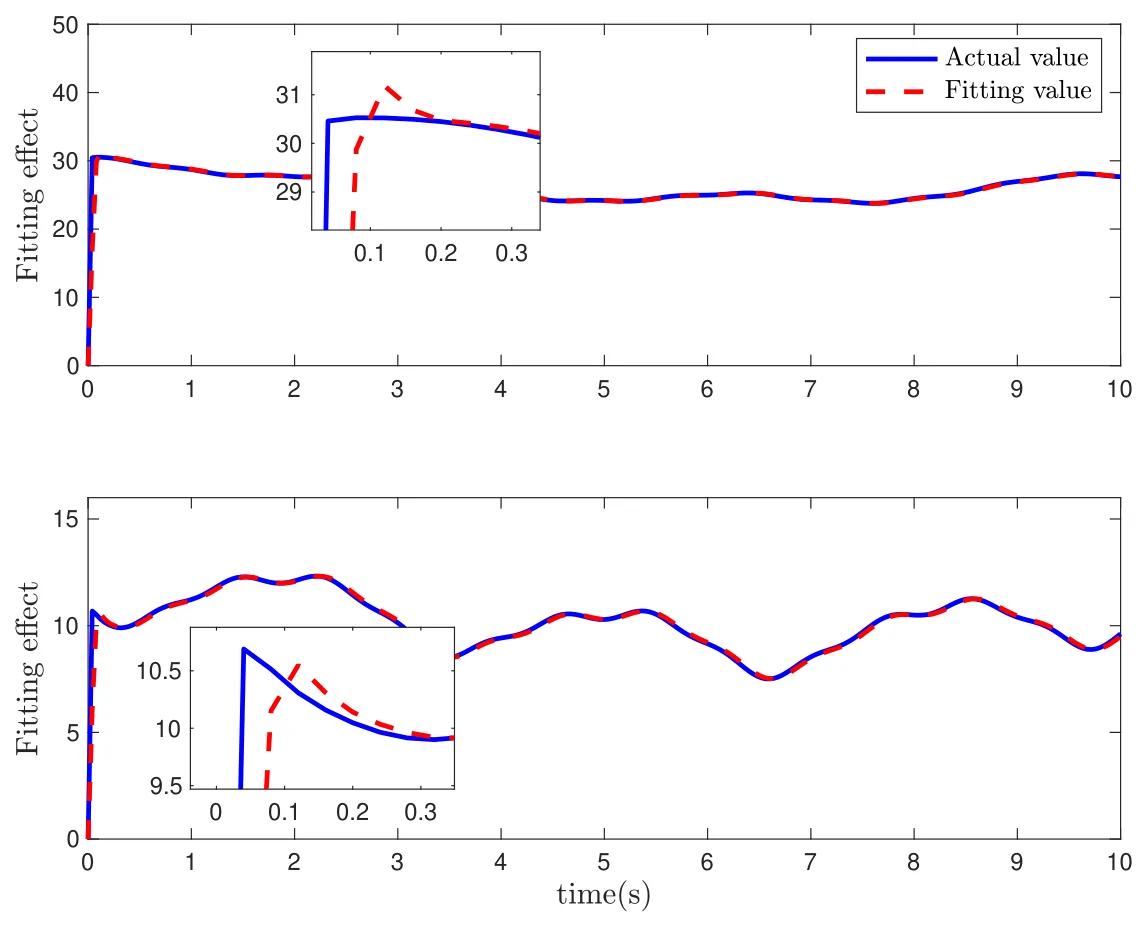
The curves of $M_{un}^{\prime }$ and ${\hat{W}}^{T}S\left(In\right)$.

**Figure 10:**
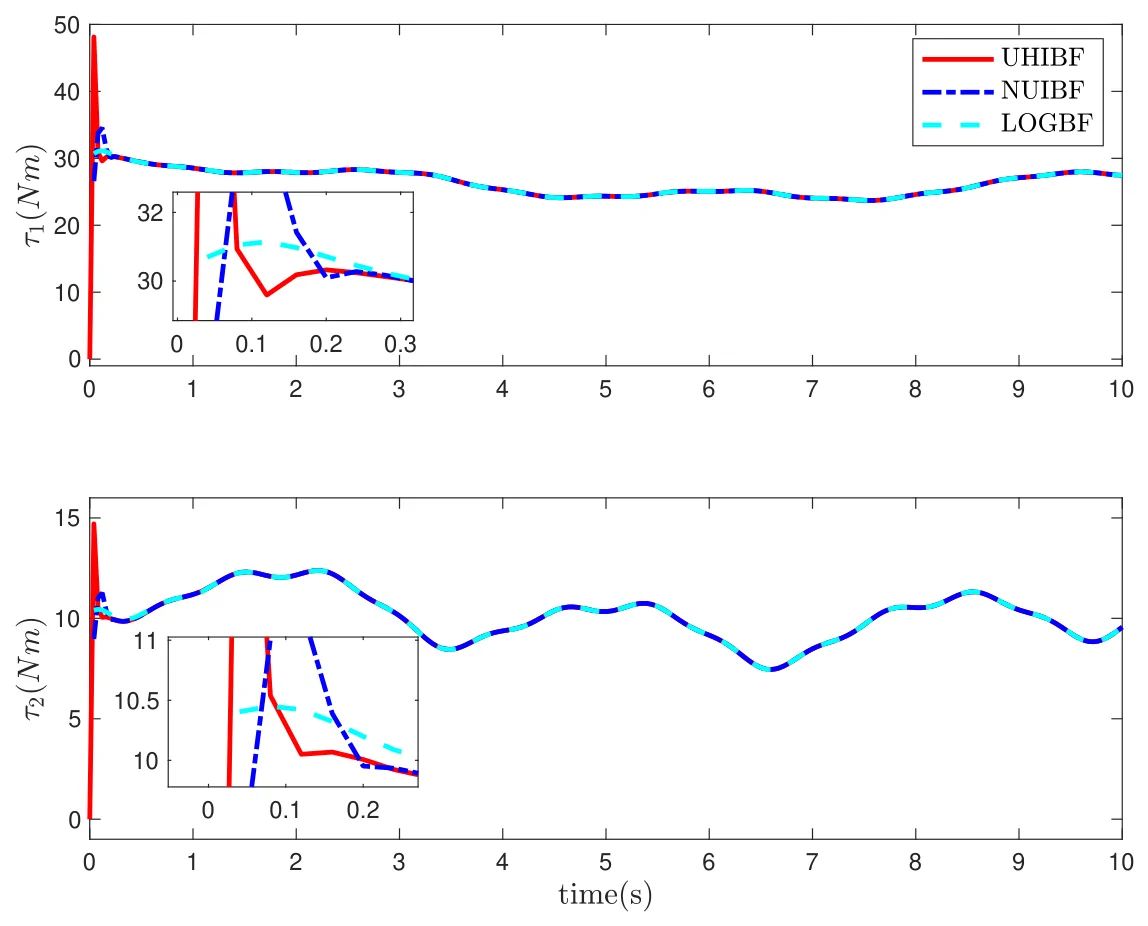
The control input τ(t).

Figure 2, Figure 3, Figure 4, Figure 5, Figure 6, Figure 7, Figure 8, Figure 9 and Figure 10 show the comparative simulation results of biomimetic rehabilitation manipulator tracking control strategies constructed with UHIBF, NUIBF and LOGBF. It can be observed from Figure 2, Figure 3, Figure 4 and Figure 5 that all three control strategies enable the manipulator to follow the preset trajectory successfully. Nevertheless, observation from the initial response stage shows that the proposed method achieves higher tracking accuracy and faster convergence. It needs to be clarified that both the UHIBF and LOGBF strategies employ neural networks to approximate and compensate for model uncertainties, while the NUIBF scheme is simulated on the premise that the manipulator dynamic model is completely known. On one hand, this result verifies the reliability of bio-inspired radial basis function neural networks, which can achieve high-precision tracking performance under the influence of model uncertainties. On the other hand, it reflects the favorable performance of the parameter-adjustable UHIBF constructed in this work. In addition, the controllers designed with UHIBF, NUIBF and traditional LOGBF all keep joint tracking errors $z_{1i}$ confined within the preset time-varying constraint boundaries throughout the simulation. None of the three schemes causes constraint violation, and this characteristic can provide theoretical support for the safe operation of the rehabilitation manipulator.

The velocity tracking errors $z_{2i}$ are depicted in Figure 6. Among the three control schemes of UHIBF, NUIBF and LOGBF, the velocity error under the UHIBF-based controller τ(t) achieves a faster convergence rate and generates slight transient overshoot. This small overshoot comes along with the fast convergence of position tracking errors. Meanwhile, the corresponding control input τ(t) delivers a larger amplitude at the initial stage. Different from the comparative control schemes, the UHIBF- based controller introduces extra tunable parameters, namely the order *q* and coefficient *p* of the integral barrier function. These two adjustable parameters play an auxiliary role in tuning the overall control performance. Figure 7 and Figure 8 illustrate the simulation results when bio-inspired radial basis function NNs are adopted to approximate the system uncertainties. Figure 8 shows the variation in the fitting residual. The simulation results reveal that the fitting residual converges rapidly to zero, delivering satisfactory approximation performance. Figure 7 displays the norm curve of bio-inspired radial basis function NN weights, while Figure 9 presents the fitting effect when the bio-inspired radial basis function NN approximates the true value of the system uncertainties. From Figure 7 and Figure 9, the weight norm evolves consistently with the fitting effect curve of the bio-inspired radial basis function NN. In the early simulation stage, the velocity error $z_{2i}$ is large and dominates the adaptive law, leading to a gradual growth of the weight norm. In steady state, $z_{2i}$ falls to a small magnitude. However, the σ-modified damping term ensures that the weight derivative never strictly equals zero. Hence, the weight norm oscillates near a steady value. This consistent trend demonstrates that the weight updating rate is determined by the real-time velocity error. As illustrated in Figure 10, the control torques of UHIBF, NUIBF and LOGBF present clear differences in the transient stage. When the system reaches the steady state, the three curves gradually coincide.

## 5. Conclusions

This study addresses the model uncertainty and position error constraint problems existing in trajectory tracking control of biomimetic rehabilitation manipulators. Different from traditional integral barrier Lyapunov functions, a UHIBF is coined in this work, which is applicable to both time-varying and time-invariant constraints for biomimetic rehabilitation manipulators. This method simplifies the controller design and maintains the differentiability of virtual control laws in backstepping control. Combined with bio-inspired radial basis function NNs, the control strategy mitigates the adverse effects of model uncertainties on rehabilitation manipulator control performance. The derived barrier function theorems ensure that all constrained errors stay within predefined boundaries and converge exponentially to a small neighborhood of zero. Theoretical analysis also verifies the exponential stability of the closed-loop system, confirming that the devised method can effectively avoid constraint violations during bionic rehabilitation robot motion tasks. Through comparative simulation experiments with the previous NUIBF-based manipulator tracking control strategy, the effectiveness of the UHIBF-based tracking control method is further validated. Meanwhile, the bio-inspired radial basis function NNs in this work are used to approximate unknown system dynamics for the case of incomplete model information. Even compared with the NUIBF-based control strategy implemented under fully known system models, our proposed method delivers better tracking performance. However, the proposed control scheme contains multiple tunable parameters; adjusting a single parameter such as *p* exerts negligible influence on overall control performance. Future research will reformulate the symmetric high-order integral barrier function developed herein into an asymmetric structure by using piecewise functions, to accommodate complex asymmetric constraint scenarios encountered in practical bionic rehabilitation robots, and corresponding verification simulations will be conducted in future investigations.

## Data Availability

The data presented in this study are available on request from the corresponding author due to internal research data management restrictions. The full raw datasets are temporarily not suitable for open public access. All valid requests for research data will be properly processed via email correspondence.

## Abbreviations

The following abbreviations are used in this manuscript:

NNs	Neural networks
UHIBF	Universal high-order integral barrier function
NUIBF	Non-universal integral barrier function
